# Specifications of the ACMG/AMP variant curation guidelines for the analysis of germline *PALB2* sequence variants

**DOI:** 10.1016/j.ajhg.2026.03.015

**Published:** 2026-03-26

**Authors:** Marcy E. Richardson, Megan F.H. Bishop, Megan A. Holdren, Miguel de la Hoya, Amanda B. Spurdle, Sean V. Tavtigian, Terra Brannan, Colin C. Young, Lauren Zec, Susan Hiraki, Clare Turnbull, Marc Tischkowitz, Kara A. Bernstein, Jean-Yves Masson, Shannon M. McNulty, Tina Pesaran, Alvaro N. Monteiro, Logan C. Walker, William D. Foulkes, Fergus J. Couch

In Table 1, under BP7, we have erroneously labeled this as “Not Used” when, in fact, it should have read “Used.” In addition, in the third column, the last bullet indicates that “note: BP4 splice predictions may not be used in conjunction with BP7_Varianle(RNA).” This is incorrect as these codes may, in fact, be used together. Therefore, we have removed both “Not” in column one and the entire clause in the last bullet of column 3.

The authors regret this error.

